# Neonatal Treatment With Beta-Cell Stimulatory Agents Reduces the Incidence of Diabetes in BB Rats

**DOI:** 10.1155/EDR.2000.1

**Published:** 2000

**Authors:** Karsten Buschard, Troels Bock, Charlotte R. Pedersen, Susanne V. Hansen, Kim Aaen, Merete JØrgensen, Michael WØllike Hansen, Troels W. Kjaer, Ida Hageman, Knud Josefsen

**Affiliations:** 1 Bartholin Instituttet Kommunehospitalet Copenhagen K DK-1399 Denmark

## Abstract

The aim of the study was to investigate whether various beta-cell stimulatory drugs, given neonatally, influence the incidence of diabetes in BB rats. Newborn BB rats were treated twice daily for 6 days and diabetes development was observed during the following 200-day study period. Compared to a diabetes incidence of 63.8% in 163 control BB rats which received saline or were untreated, the percentage of experimental BB rats that developed diabetes was as follows in the different subgroups: arginineglucose: 47% (*n*= 73, *p* < 0.02); glucagon: 37% (*n* = 93, *p* <  0.0001); tolbutamide-glucose: 36% (*n* = 58, *p* < 0.0005); and theophylline-glucose: 39% (*n* = 41, *p* < 0.005). A long-term arginine-glucose treatment was not superior to the shorter neonatal treatment. Histological examination revealed a higher degree of insulitis in diabetic than in non-diabetic animals but no difference according to the kind of treatment was observed. Finally, we found that the diabetes incidence in BB rats was higher in the first litter compared to subsequent litters (*p* = 0.04). Thus, neonatal treatment with various beta-cell stimulatory agents reduces diabetes incidence in BB rats. The theory behind the study, that the treatment accelerates beta-cell maturation leading to increased immunological tolerance towards beta cells, is discussed.


Int. Jnl. Experimental Diab. Res., Vol. 1, pp. 1–8
Reprints available directly from the publisher
Photocopying permitted by license only

© 2000 OPA (Overseas Publishers Association) N.V.
Published by license under
the Harwood Academic Publishers imprint,
part of The Gordon and Breach Publishing Group.
Printed in Malaysia.

### Neonatal Treatment with Beta-cell Stimulatory Agents Reduces the Incidence of Diabetes in BB Rats

KARSTEN BUSCHARD*, TROELS BOCK, CHARLOTTE R. PEDERSEN, SUSANNE V. HANSEN, KIM AAEN, MERETE JØRGENSEN, MICHAEL WØLLIKE HANSEN, TROELS W. KJAER, IDA HAGEMAN and KNUD JOSEFSEN

*Bartholin Instituttet, Kommunehospitalet, DK-1399 Copenhagen K, Denmark*

*(Received 20 June 1999; In final form 8 August 1999)*

**The aim of the study was to investigate whether various beta-cell stimulatory drugs, given neonatally, influence the incidence of diabetes in BB rats. Newborn BB rats were treated twice daily for 6 days and diabetes development was observed during the following 200-day study period. Compared to a diabetes incidence of 63.8% in 163 control BB rats which received saline or were untreated, the percentage of experimental BB rats that developed diabetes was as follows in the different subgroups: arginine-glucose: 47% ($n = 73$, $p < 0.02$); glucagon: 37% ($n = 93$, $p < 0.0001$); tolbutamide-glucose: 36% ($n = 58$, $p < 0.0005$); and theophylline-glucose: 39% ($n = 41$, $p < 0.005$). A long-term arginine-glucose treatment was not superior to the shorter neonatal treatment. Histological examination revealed a higher degree of insulitis in diabetic than in non-diabetic animals but no difference according to the kind of treatment was observed. Finally, we found that the diabetes incidence in BB rats was higher in the first litter compared to subsequent litters ($p = 0.04$). Thus, neonatal treatment with various beta-cell stimulatory agents reduces diabetes incidence in BB rats. The theory behind the study, that the treatment accelerates beta-cell maturation leading to increased immunological tolerance towards beta cells, is discussed.**

*Keywords:* Neonatal, beta-cell stimulation, diabetes, BB rats, tolbutamide, theophylline

### INTRODUCTION

The idea of neonatal stimulation of beta cells in BB rats and NOD mice in order to reduce the diabetes incidence (Buschard *et al.*, 1990) is inspired by two observations.

Firstly, Type 1 diabetes is less frequent in children of Type 1 diabetic mothers than in children of diabetic fathers (Warram *et al.*, 1984; Tilli and Köbberling, 1987; Rjasanowski *et al.*, 1990); in other words: a Type 1 diabetic pregnancy seems to partially protect against development of Type 1 diabetes in the offspring. Even if the mothers develop Type 1 diabetes during pregnancy, diabetes is infrequent among the children (Buschard *et al.*, 1989). Interestingly, a growing knowledge indicates that if the mothers develop Type 1 diabetes after the pregnancy, the children seem to have the same diabetes risk as children of diabetic fathers (Warram *et al.*, 1984; Lorentzen *et al.*, 1998).

Secondly, antigen expression of the beta cells seems to be dependent on their functional state; at a high function there is a high expression. This

*Corresponding author. Fax: +45-33 93 85 66, e-mail: buschard@dadlnet.dk has been found for the antigens corresponding to the (monoclonal) antibodies IC2 (Buschard *et al.*, 1988; Aaen *et al.*, 1990), A2B5 (Aaen *et al.*, 1990; Eisenbarth *et al.*, 1982), R2D6 (Appel *et al.*, 1989), and ICA (McCulloch *et al.*, 1991), and for GAD (Hao *et al.*, 1994) and 64 kDa antigen (Kämpe *et al.*, 1989).

Normally, beta cells are immature at birth (Freinkel *et al.*, 1984; Grasso *et al.*, 1973; Otonkoski *et al.*, 1988) whereas in the diabetic pregnancy the beta cells mature earlier (Heding *et al.*, 1980). Since diabetes is an autoimmune disease resulting from a break of self tolerance, we wanted to investigate whether neonatal beta-cell stimulation, and thereby early maturation of the beta cells like in the diabetic pregnancy with fetal hyperglycemia, could lower the diabetes incidence. Normally, neonatal beta cells are insensitive to glucose during the first two weeks of life (Otonkoski *et al.*, 1988) but sensitive to arginine, glucagon, and theophylline (Otonkoski, 1988). Therefore, in this study we used the three mentioned agents as well as tolbutamide; all these molecules are non-toxic to mammalians and they have different mechanisms of action. The optimal duration of the treatment is unknown. Therefore, besides short-term stimulation which we arbitrarily chose to be twice daily for six days, we also performed a prolonged experiment with treatment weekly throughout the entire study.

### METHODS

### Neonatal Stimulation of Beta Cells

BB rats (Møllegaard, Ll. Skensved, Denmark) were paired in our Institute. No diabetic BB rats were used as fathers, or as mothers during the pregnancy and the weaning period. From the day of birth and during the 5 following days the newborn rats were treated twice daily (morning and evening) with intraperitoneal or subcutaneous injections of 100 µl sterile water or saline to which had been added: 7.5 mg (1.1 g/kg body weight) L-arginine (Sigma, St. Louis, USA) and 2.5 mg (0.4 g/kg BW) glucose ($n = 73$ BB rats); 0.01 mg (1.4 mg/kg BW) glucagon (Novo Nordisk, Bagsvaerd, Denmark) and 5 mg (0.7 g/kg BW) glucose ($n = 43$); 0.02 mg (2.8 mg/kg BW) glucagon zinc protamine (Novo Nordisk) ($n = 50$); 1.25 mg (180 mg/kg BW) tolbutamide (Hoechst, Frankfurt, Germany) and 10 mg (1.4 g/kg BW) glucose ($n = 58$); or 0.140 mg (20 mg/kg BW) theophylline and 10 mg (1.4 g/kg BW) glucose ($n = 41$). The dosage of the different compounds was arbitrarily chosen within the limits of possible pharmacological treatment. Addition of glucose was performed in order to avoid any risk of hypoglycemia and the amount was arbitrary as well. Control BB rats were given saline i.p. ($n = 46$), saline s.c. ($n = 49$), or were untreated ($n = 68$).

The animals were weaned after 3 weeks and allowed free access to chow and water. The animals were observed daily, and weighed and examined weekly for glucosuria (TesTape, Lilly, Indianapolis, USA). Diabetes was diagnosed if at least ++ glucosuria (1/4%) with >10% weight loss (compared to the previous week) was observed. When this occurred the animals were sacrificed by $CO_2$ anesthesia and the blood glucose values were measured. The study was terminated at day 200.

In a long-term arginine-glucose treatment group ($n = 31$), L-arginine (75 mg/ml)-glucose (25 mg/ml) was given i.p. twice a day from the day of birth and during the 5 following days, afterwards once a week to the end of the study at a dose of 100 µl/6 g BW up to a maximum dose of 1000 µl. Control BB rats ($n = 36$) were correspondingly given saline.

### Histological Studies

The paraffin-embedded pancreases were cut into 4 µm sections and stained with hematoxylin and eosin. An area of 11.4 mm$^2$ (11,400,000 µm$^2$)

in sections from each BB rat was scored in a Leitz Wetzler dialux microscope equipped with a Microvid image analysis system for the degree of insulitis. Insulitis was scored semiquantitatively with *Grade* 0 denoting morphologically unaffected islets, *Grade 1* a very disperse intra-insular infiltration of mononuclear cells, *Grade 2* a more pronounced intra-insular infiltration with either disperse or small clusters of mononuclear cells and, finally, *Grade 3* denoting islets dominated by infiltrating mononuclear cells. In Grade 3 the islets often show an altered architecture and pyknotic cell nuclei. Islets with peri-insular infiltration were placed in group 2 or 3 depending on the intra-insular infiltration of mononuclear cells. Ten islets were scored in each section and the average was calculated and considered the final score of insulitis.

### Statistics

The cumulative diabetes incidence observed in each test group was calculated by Kaplan-Meier estimation and statistical significance was evaluated by the log-rank (Peto) test. Differences in diabetes incidence between groups of litters were compared by means of t-test after a variance stabilizing angle transformation of the frequency within litters (Hald, 1957). Other differences were estimated by the Mann-Whitney test. Spearman's rank correlation test was used for the calculations of the coefficient of correlation. Data are presented as mean $\pm$ SEM.

### RESULTS

### Diabetes Incidence

The results of the different neonatal treatments can be seen in Figure 1 and Table I. The experimental BB rats received either arginine, glucagon, tolbutamide or theophylline, and the diabetes incidences were found to remain between 30% and 47%. There were no differences between the various treated groups, and their incidence curves were comparable. On the other hand, all the experimental groups each displayed a substantially lower diabetes incidence than the 163 control BB rats of which 63.8% developed diabetes during the 200 days observation period.

In Figure 2 the rats are divided according to sex. The curves for the male and female rats were similar for both the treated and control animals, respectively, apart from a slightly lower incidence of diabetes for the female BB rats at the end of the study (treated BB rats: 41.1% *vs.* 38.6%, $p > 0.10$, control BB rats: 67.6% *vs.* 60.9%, $p > 0.10$).

Figure 3 shows the result of the extended arginine-glucose treatment. The diabetes incidence was significantly lower ($p = 0.04$) for the long-term treated animals than for the control group.

### Histological Studies

The result of the examination of the islets for insulitis is shown in Table II. All the groups of diabetic rats had significantly higher scores than the non-diabetic rats. There were no differences between the various experimental groups. However, it should be noticed that overall the degree of insulitis was modest and massive insulitis was only rarely seen. Control rats with early diabetes development (before day 100) showed similar degrees of insulitis as rats that became diabetic after day 100 ($1.4 \pm 0.1$ *vs.* $1.1 \pm 0.1$, ($p > 0.10$). Likewise, no differences were seen for treated diabetic animals ($1.2 \pm 0.2$ *vs.* $1.1 \pm 0.1$ insulitis score).

### Studies of Litters and Sex

Litters contained more female than male BB rats in both the experimental and control groups (42.3% and 43.6% males, respectively). Litter sizes were similar in the experimental and the

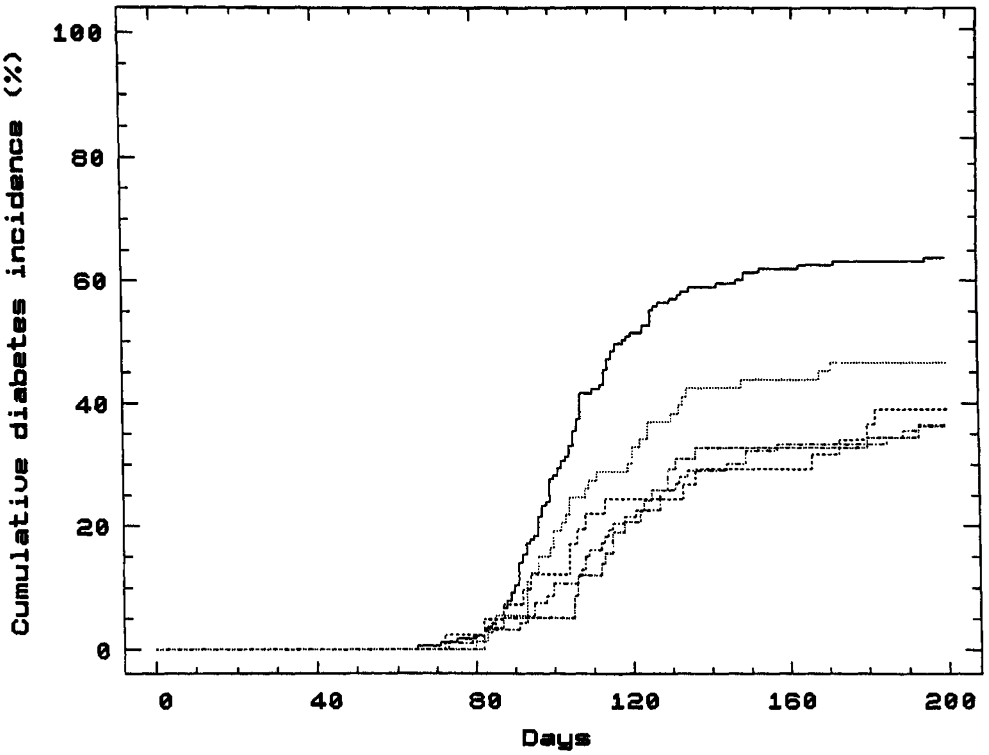

FIGURE 1 Cumulative diabetes incidence for the different treated groups (arginine-glucose ....; glucagon ----; tolbutamide-glucose ----; theophylline-glucose ----) and control group of BB rats (—).

TABLE I Number of animals and cumulative diabetes incidence at 200 days of age in the different treatment groups

| | Number of BB rats | Diabetes incidence at 200 days of age (%) | Difference from controls |
|---|---|---|---|
| Arginine-glucose | 73 | 47 | $p<0.02$ |
| Glucagon-glucose | 43 | 42 | $p<0.02$ |
| Glucagon zink protamine | 50 | 30 | $p<0.00005$ |
| Tolbutamide-glucose | 58 | 36 | $p<0.0005$ |
| Theophylline-glucose | 41 | 39 | $p<0.005$ |
| Controls | 163 | 64 | – |

control group (6.6 ± 0.5 and 6.5 ± 0.7 rats, respectively). There was no association between litter size and diabetes incidence in any of the groups. Furthermore, the mean litter size was the same in the different litter generations.

To investigate the correlation between litter number and diabetes incidence, the 25 control litters with a total of 163 offspring were divided into a group of 12 first litters and a group of 13 second or later litters (Tab. III). The overall diabetes incidences were 57/77 = 0.74 and 47/86 = 0.54, respectively. By comparison of these incidences after angle transformation the difference was found statistically significant ($p = 0.04$). The mean litter number was 1.75 ± 0.20 for the experimental BB rats and 1.96 ± 0.24 for the controls, which was not significantly different.

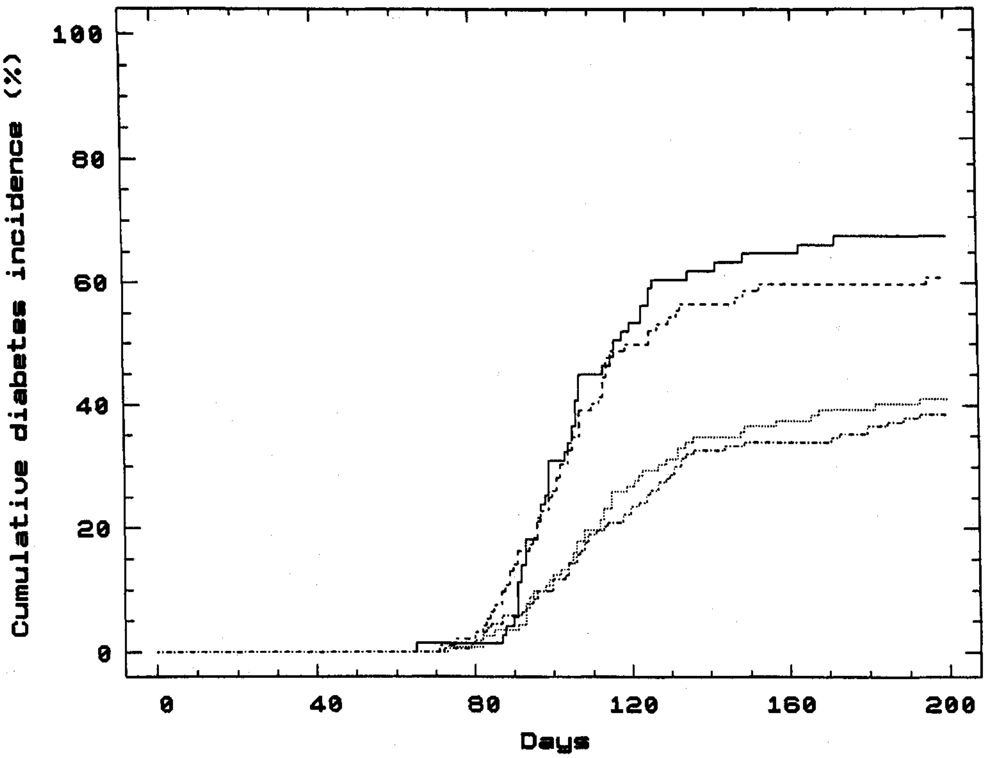

FIGURE 2 Cumulative diabetes incidence for male and female BB rats in the collective neonatally treated group (M . . . . ; F·--·) and in the control group (M —; F ---).

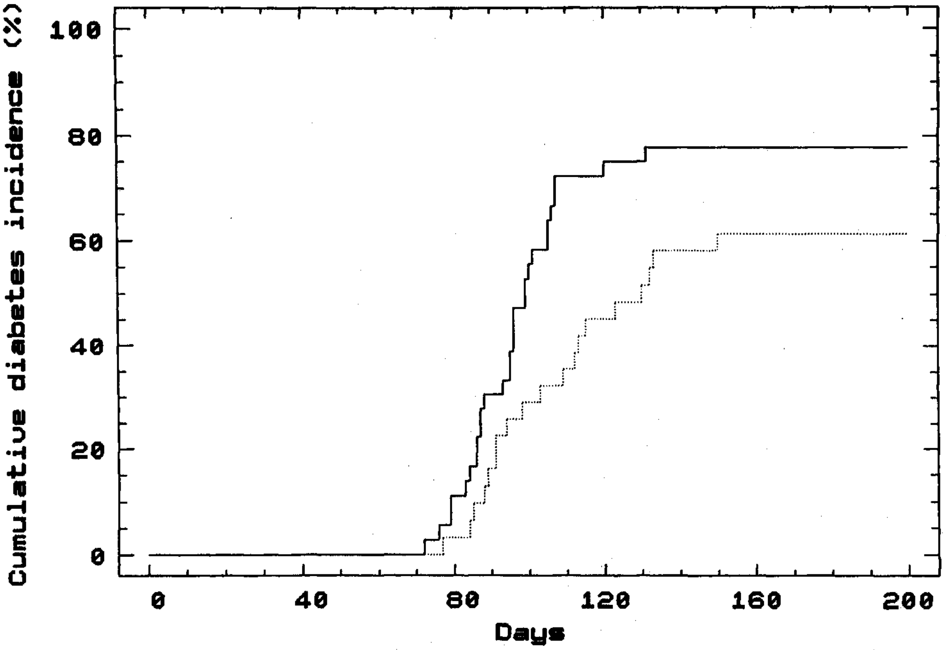

FIGURE 3 Cumulative diabetes incidence for long-term arginine-glucose treated BB rats (. . . .) and their saline treated controls (—).

TABLE II Average degree of insulitis ±SEM in the different groups. Number of investigated animals is indicated in brackets. Significance tests refer to the result of non-diabetic control BB rats

| | Non-diabetic rats | Diabetic rats |
|---|---|---|
| Control BB rats | 0.6±0.1 (70) | 1.2±0.1 (87) $p<10^{-7}$ |
| Treated BB rats | 0.8±0.1 (158) $p<0.005$ | 1.1±0.1 (88) $p<10^{-6}$ |
| Long-term treated BB rats | 0.7±0.2 (9) n.s. | 1.1±0.2 (15) $p<0.005$ |

TABLE III Litter size and diabetes incidence for the first and second-fifth litter numbers, respectively, among the diabetic control BB rats

| Litter number | Litter size diabetes incidence (%) | | | | | | | | | | | | |
|---|---|---|---|---|---|---|---|---|---|---|---|---|---|
| First | 2 | 2 | 7 | 5 | 2 | 10 | 10 | 3 | 6 | 13 | 9 | 8 | |
| | 50 | 50 | 86 | 80 | 50 | 50 | 80 | 67 | 67 | 69 | 89 | 100 | |
| Second-fifth | 9 | 11 | 8 | 6 | 6 | 3 | 3 | 11 | 13 | 4 | 3 | 4 | 5 |
| | 67 | 45 | 88 | 67 | 83 | 33 | 67 | 64 | 54 | 50 | 0 | 0 | 20 |

### DISCUSSION

The present investigations have shown in large-scale experiments that the diabetes incidence in BB rats can be reduced by neonatal injections of various agents known to stimulate insulin secretion by different mechanisms. These agents include arginine, glucagon, and for the first time tolbutamide and theophylline. Also, for long-term treatment with arginine-glucose a reduction in diabetes incidence is found, but this treatment is not superior compared with the proper neonatal one.

Until now only a few other studies have dealt with the concept of neonatal beta-cell stimulation. In a barrier-confined colony of BB rats with a high diabetes incidence, this was moderately but significantly reduced and the onset of diabetes delayed after neonatal treatment with arginine and glucose combined, but given only once a day (Hansen *et al.*, 1993). In NOD mice the diabetes incidence has been found by our group to be reduced by neonatal treatment with glucose (Bock *et al.*, 1991). We have as yet no explanation for the discrepancy between NOD mice and BB rats in which glucose treatment *per se* does not significantly alter the diabetes incidence (Buschard *et al.*, 1990). Interestingly, female NOD mice treated with neonatal injections the first 6 days of life with 1 mg arginine and 0.5 mg glucose combined displayed diabetes earlier and with a higher incidence (Senecat *et al.*, 1994). Obviously, the outcome of arginine on the one hand and of pure glucose on the other is different, and the effect of these compounds on beta-cell maturation should be studied with molecular biological methods. In another approach of early beta-cell stimulation, 4-week old NOD female mice were fed with 1 to 1.75 g glucose orally per day until the age of 30 weeks; at that time 37% of the experimental mice had developed diabetes compared to 86% of the controls (Vardi and Buschard, 1995). Regarding tolbutamide, treatment starting at the time of weaning has resulted in a reduced diabetes incidence in NOD mice (Williams *et al.*, 1993); although different in time of treatment this study may be comparable with our neonatal one.

The precise mechanism behind the observed reduction of diabetes incidence and delayed onset of the disease in BB rats is unknown. The theory behind the study is that the antigenic self of the beta cells is less well established because the cells are immature at birth and not fully developed until later in life; antigenic differences should then exist between young and adult beta cells, which has indeed been confirmed. A 38 kDa protein, reacting with sera from diabetic BB rats, is present in adult but not in neonatal BB rat islet cells (Ko *et al.*, 1994).

Furthermore, adult but not neonatal islets are destroyed after transplantation to diabetic BB rats (Ihm *et al.*, 1991). Another study measured *in vitro* cytotoxicity mediated by mononuclear spleen cells from newly diabetic BB rats and found a significantly lower cytotoxicity towards islet cells from BB rats < 4 days old than towards islet cells from adult insulitis-free BB rat islets (Ekblond *et al.*, 1995). Islet cell maturation in BB rats – as evidenced by sensitivity to cytotoxicity – was not seen before the age of 21 days after birth (Ekblond *et al.*, 1995).

The finding in the present study – that the litter sequence number seems to influence the diabetes incidence in BB rats – is to our knowledge a new observation which may reflect that, among diabetes-prone rats, the risk of developing diabetes in the offspring decreases with maternal age; this parallels findings in humans (Warram *et al.*, 1991).

It has been suggested that the protection of the diabetic pregnancy against development of diabetes in the offspring may be due to a direct immunological mechanism (Dosch *et al.*, 1993). This might be mediated by placental transfer of autoantibodies which in the fetus, according to the immunological network theory, might raise specific regulator cells. However, offspring of Type 2 diabetic mothers also have lower incidence of Type 1 diabetes compared to children of Type 2 diabetic fathers (Green *et al.*, 1994) and Type 2 diabetic mothers do not display autoantibodies. This may support the mechanism involving early stimulation of the beta-cells as suggested in this study.

### *Acknowledgements*

We thank Aage Vølund PhD, Novo Nordisk, Bagsvaerd, Denmark, for statistical help.

### *References*

Aaen, K., Rygaard, J., Josefsen, K., Petersen, H., Brogren, C.-H., Horn, T. and Buschard, K. (1990) Dependence of antigen expression on functional state of beta-cells. *Diabetes*, **39**, 697 – 701.

Appel, M. C., Dotta, F., O'Neil, J. J. and Eisenbarth, G. S. (1989) B-cell activity regulates the expression of islet antigenic determinants. *Diabetologia*, **32**, 461A.

Bock, T., Kjaer, T. W., Jørgensen, M., Josefsen, K., Rygaard, J. and Buschard, K. (1991) Reduction of diabetes incidence in NOD mice by neonatal glucose treatment. *APMIS*, **99**, 989 – 992.

Buschard, K., Brogren, C.-H., Röpke, C. and Rygaard, J. (1988) Antigen expression of the pancreatic beta-cells is dependent on their functional state, as shown by a specific, BB rat monoclonal autoantibody IC2. *APMIS*, **96**, 342 – 346.

Buschard, K., Kühl, C., Mølsted-Pedersen, L., Lund, E., Palmer, J. and Bottazzo, G. F. (1989) Investigation in children who were *in utero* at onset of insulin-dependent diabetes in their mothers. *Lancet*, **i**, 811 – 814.

Buschard, K., Jørgensen, M., Aaen, K., Bock, T. and Josefsen, K. (1990) Prevention of diabetes mellitus in BB rats by neonatal stimulation of beta cells. *Lancet*, **335**, 134 – 135.

Dosch, H.-M., Martin, J. M., Robinson, B. H., Åkerblom, H. K. and Karjalainen, J. (1993) An immunological basis for disproportionate diabetes risk in children with a type I diabetic mother or father. *Diabetes Care*, **16**, 949 – 951.

Eisenbarth, G. S., Shimizu, K., Bowring, M. A. and Wells, S. (1982) Expression of receptors for tetanus toxin and monoclonal antibody A2B5 by pancreatic islet cells. *Proc. Natl. Acad. Sci. USA*, **79**, 5066 – 5070.

Ekblond, A., Schou, M. and Buschard, K. (1995) Cytotoxicity towards neonatal *versus* adult BB rat pancreatic islet cells. *Autoimmunity*, **20**, 93 – 95.

Freinkel, N., Lewis, N. J., Johnson, R., Swenne, I., Bone, A. and Hellerström, C. (1984) Differential effects of age *versus* glycemic stimulation on the maturation of insulin stimulus-secretion coupling during culture of fetal rat islets. *Diabetes*, **33**, 1028 – 1038.

Grasso, S., Messina, A., Distefano, G., Vigo, R. and Reitano, G. (1973) Insulin secretion in the premature infant. *Diabetes*, **22**, 349 – 353.

Green, A. and the EURODIAB ACE Study Group (1994) Geographic variation in the recurrence risk in close relatives of childhood onset IDDM patients. *Diabetologia*, **37**, suppl. 1, A6.

Hald, A. (1957) *Statistical theory with engineering applications*. New York: John Wiley & Sons, p. 685.

Hansen, A. K., Josefsen, K., Pedersen, C. and Buschard, K. (1993) Neonatal stimulation of beta-cells reduces the incidence and delays the onset of diabetes in a barrier-protected breeding colony of BB rats. *Exp. Clin. Endocrinol.*, **101**, 189 – 193.

Hao, W., Li, L., Mehta, V., Lernmark, A. and Palmer, J. P. (1994) Functional state of the beta cell affects expression of both forms of glutamic acid decarboxylase. *Pancreas*, **9**, 558 – 562.

Heding, L. G., Persson, B. and Stangenberg, M. (1980) B-cell function in newborn infants of diabetic mothers. *Diabetologia*, **19**, 427 – 432.

Ihm, S.-H., Lee, K.-U. and Yoon, J.-W. (1991) Studies on autoimmunity for initiation of beta-cell destruction. VII. Evidence for antigenic change on beta cells leading to autoimmune destruction of beta cells in BB rats. *Diabetes*, **40**, 269 – 274.

Kämpe, O., Andersson, A., Björk, E., Hallberg, A. and Karlsson, F. A. (1989) High-glucose stimulation of 64,000-$M_r$ islet cell autoantigen expression. *Diabetes*, **38**, 1326 – 1328.

Ko, I. Y., Jun, H. S., Kim, G. S. and Yoon, J.-W. (1994) Studies on autoimmunity for initiation of beta-cell destruction. X. Delayed expression of a membrane-bound islet cell-specific 38 kDa autoantigen that precedes insulitis and diabetes in the diabetes-prone BB rat. *Diabetologia*, **37**, 460–465.

Lorentzen, T., Pociot, F., Stilgren, L., Kristiansen, O. P., Johannesen, J., Olsen, P. B., Walmer, A., Larsen, A., Albrechtsen, N. C., Eskildsen, P. C., Andersen, O. O., Nerup, J. and the Danish IDDM Epidemiology and Genetics Group (1998) Predictors of IDDM reoccurrence risk in offspring of Danish IDDM patients. *Diabetologia*, **41**, 666–673.

McCulloch, D. K., Barmeier, H., Neifing, J. L. and Palmer, J. P. (1991) Metabolic state of the pancreas affects end-point titre in the islet cell antibody assay. *Diabetologia*, **34**, 622–625.

Otonkoski, T. (1988) Insulin and glucagon secretory responses to arginine, glucagon, and theophylline during perfusion of human fetal islet-like cell clusters. *J. Clin. Endocrinol. Metab.*, **67**, 734–740.

Otonkoski, T., Andersson, S., Knip, M. and Simell, O. (1988) Maturation of insulin response to glucose during human fetal and neonatal development. *Diabetes*, **37**, 286–291.

Rjasanowski, I., Heinke, P., Michaelis, D. and Kurajewa, T. L. (1990) The higher frequency of type I (insulin-dependent) diabetes in fathers than in mothers of type I-diabetic children. *Exp. Clin. Endocrinol.*, **95**, 91–96.

Senecat, O., Martignat, L., Elmansour, A., Charbonnel, B. and Sai, P. (1994) Diabetes enhancement and increased islet antigen expression following neonatal injections of glucose and arginine in Non-Obese Diabetic mice. *Metabolism*, **43**, 1410–1418.

Tilli, H. and Köbberling, J. (1987) Age-corrected empirical genetic risk estimates for first-degree relatives of IDDM patients. *Diabetes*, **36**, 93–99.

Vardi, P. and Buschard, K. (1995) Impact of early pancreatic islet stimulation on the natural history of insulin-dependent diabetes. In: Shafrir, E. (Ed.) *Lessons from animal diabetes V* (London: John Libbey), pp. 35–40.

Warram, J. H., Krolewski, A. S., Gottlieb, M. S. and Kahn, C. R. (1984) Differences in risk of insulin-dependent diabetes in offspring of diabetic mothers and diabetic fathers. *N. Engl. J. Med.*, **311**, 149–152.

Warram, J. H., Martin, B. C. and Krolewski, A. S. (1991) Risk of IDDM in children of diabetic mothers decreases with increasing maternal age at pregnancy. *Diabetes*, **40**, 1679–1680.

Williams, A. J., Beales, P. E., Krug, J., Procaccini, E., Signore, A., Xu, S., Gale, E. A. and Pozzilli, P. (1993) Tolbutamide reduces the incidence of diabetes mellitus, but not insulitis, in the non-obese-diabetic mouse. *Diabetologia*, **36**, 487–492.

